# Editorial: Genetic approaches for crop yield enhancement

**DOI:** 10.3389/fpls.2023.1176522

**Published:** 2023-03-24

**Authors:** Dongying Gao, Guo-qing Song, Ahmad A. Omar

**Affiliations:** ^1^ Small Grains and Potato Germplasm Research Unit, United States Department of Agriculture-Agricultural Research Service (USDA-ARS), Aberdeen, ID, United States; ^2^ Plant Biotechnology Resource and Outreach Center, Department of Horticulture, Michigan State University, East Lansing, MI, United States; ^3^ Citrus Research and Education Center, University of Florida, Institute of Food and Agricultural Sciences (IFAS), Lake Alfred, FL, United States; ^4^ Biochemistry Department, Faculty of Agriculture, Zagazig University, Zagazig, Egypt

**Keywords:** yield, genetic improvement, annual and perennial crops, QTL, molecular mapping

The world food safety faces numerous challenges, such as growing population, decreasing natural resources, and global warming. Experts predict that we will not be able to grow enough food to feed the world’s population by 2050 unless the yield increases significantly. Thus, efforts to increase the crop production remain a top mission for all plant scientists in agriculture across the world. Among all agronomic traits, yield improvement is the most important objective for most, if not for all, crop breeding programs. The Green Revolution in 1960s, which was achieved by the improvement of plant architecture with semidwarf genes, development of hybrid varieties, application of chemical fertilizers and other practices, significantly increased crop yield and the world’s food supply (Pingali, 2012). Since then, it has been becoming extremely difficult to steeply increase yield for many major crops. The goals of this Research Topic, “Genetic Approaches for Crop Yield Enhancement”, were to collect the related manuscripts and highlight the emerging approaches for enhancing crop yield with traditional, molecular, and other newly developed strategies. Four original articles were submitted and published in this section which provide new insights into the genetic and genomic basis of yield and other important traits in different crops.

Application of fertilizers is an effective approach to increase crop yield as they provide the essential nutrient for plant growth and development. However, overuse of fertilizers can also cause environmental pollutions and affect the end-use quality of crops. Therefore, it is important to understand the global mechanisms of chemical macronutrients such as nitrogen, phosphorus and sulfur on crop yield and quality. Liu et al. investigated applications of sulfur, 0 kg·ha^−1^ versus 60 kg·ha^−1^, at three seed development stages of bread wheat (*Triticum aestivum*) and conducted transcriptomic and metabolomic comparisons. Their results showed that some MYB transcription factors (TFs) may play important roles in the process of sulfur metabolism. This provides excellent sources for identifying the key transcriptional regulators related to sulfur metabolism for wheat seed development and yield improvement.

Crop yield protection from both biotic and abiotic stresses is of great importance for food safety. Various plant pathogens and pests can cause prominently global yield losses for many staple crops, such as about 21% in wheat and 30% in rice (Savary et al., 2019). Identifying new resistant resources and understanding the genetic basis of host resistance are always helpful for enhancing crop disease resistance. For example, sugarcane (*Saccharum* spp. hybrids) smut caused by *Sporisorium scitamineum* is an important fungal disease globally. Wu et al. presented their genetic analysis of smut resistance in F1 population generated by two sugarcane parents with different levels of disease resistance. They performed bulked segregant RNA-sequence analysis (BSR-Seq) and identified 7,295 differentially expressed genes between the two pools. Interestingly, a 1.27 Mb region at chromosome Chr5B that contains the candidate genes associated with smut resistance was identified. The polymorphic SNPs and candidate genes lay a foundation for marker-assisted breeding and for further functional characterization of smut resistance gene(s) in sugarcane.

Great efforts have been made to increase biomass yield for many non-grain plants, however, the genetic basis of their biomass yield still is poorly understood. Switchgrass (*Panicum virgatum*) is one of the best renewable bioenergy crops and widely used for forages, grazing, ground cover and others. Razar et al. generated two F1 populations with three switchgrass ecotypes, lowland, upland and coastal, and conducted genotyping-by-sequencing (GBS) analysis. They found segregation distortion of alleles and constructed two genetic linkage maps. Additionally, several biomass QTLs were identified. This study provides invaluable resource for pyramiding the desirable traits including high biomass yield, cold tolerance, and non-dormancy in switchgrass breeding programs.

Annual and perennial plants are the two major types of domesticated crops in our planting systems.

The perennial crops can reduce the need for tillage, increase biological carbon sequestration, and provide many other benefits such as less soil erosion and nitrogen input. However, many major grain crops are annual that require annually replanting and cultivation. Is it possible to reduce the input costs *via* development and growth of perennial grain crops? Chapman et al. compared annual and perennial crops, reviewed the genetic and physiological basis of perennialism, and discussed the challenges of perennial grain crops, especially the transfer of some perennial traits from wild species into cultivated crops such as rice (*Oryza sativa*) and barley (*Hordeum vulgare*). Although perennial crops can bring some benefits, two major challenges are needed to be solved for perennial grain crops, including how to reach the high grain yield of the annually cultivated crops and how to reduce the losses caused by different pathogens without rotations with other crops.

In summary, yield is one of the most complex traits for crop genetic improvement, it is heavily affected by plant genotypes and numerous environmental factors. With the changing climate and increasing population, yield improvement remains the top priority of crop breeding. The four articles published in this collection open a window to show the potentials of plant breeding for yield improvement by combining traditional methods and newly evolved tools such as molecular and genomic selection and gene-editing. Apparently, genetic approaches for crop yield enhancement are desirable and achievable by manipulating many potential pathways and genes. We believe new generation varieties will make sustainable and more profitable crop productions for farmers, industries, and other related stakeholders, especially for the small-scale crop producers in the developing countries or remote regions.

## Author contributions

G-QS, AO and DG proposed the Research Topic, DG and G-QS drafted the editorial, all authors edited and approved the submission.
